# Histone acetyltransferases in rice (*Oryza sativa* L.): phylogenetic analysis, subcellular localization and expression

**DOI:** 10.1186/1471-2229-12-145

**Published:** 2012-08-15

**Authors:** Xia Liu, Ming Luo, Wei Zhang, Jinhui Zhao, Jianxia Zhang, Keqiang Wu, Lining Tian, Jun Duan

**Affiliations:** 1 South China Botanical Garden, Chinese Academy of Sciences, Guangzhou 510650, China; 2 Graduate School of the Chinese Academy of Sciences, Beijing 100039, China; 3 Southern Crop Protection and Food Research Centre, Agriculture and Agri-Food Canada, London, ON N5V 4T3, Canada; 4 Institute of Plant Biology, National Taiwan University, Taipei 106, Taiwan

**Keywords:** Histone acetyltransferase, Hormone, Phylogenetic tree, Subcellular localization, Rice, Stress

## Abstract

**Background:**

Histone acetyltransferases (HATs) play an important role in eukaryotic transcription. Eight HATs identified in rice (OsHATs) can be organized into four families, namely the CBP (OsHAC701, OsHAC703, and OsHAC704), TAF_II_250 (OsHAF701), GNAT (OsHAG702, OsHAG703, and OsHAG704), and MYST (OsHAM701) families. The biological functions of HATs in rice remain unknown, so a comprehensive protein sequence analysis of the HAT families was conducted to investigate their potential functions. In addition, the subcellular localization and expression patterns of the eight OsHATs were analyzed.

**Results:**

On the basis of a phylogenetic and domain analysis, monocotyledonous CBP family proteins can be subdivided into two groups, namely Group I and Group II. Similarly, dicotyledonous CBP family proteins can be divided into two groups, namely Group A and Group B. High similarities of protein sequences, conserved domains and three-dimensional models were identified among OsHATs and their homologs in *Arabidopsis thaliana* and maize. Subcellular localization predictions indicated that all OsHATs might localize in both the nucleus and cytosol. Transient expression in *Arabidopsis* protoplasts confirmed the nuclear and cytosolic localization of OsHAC701, OsHAG702, and OsHAG704. Real-time quantitative polymerase chain reaction analysis demonstrated that the eight *OsHATs* were expressed in all tissues examined with significant differences in transcript abundance, and their expression was modulated by abscisic acid and salicylic acid as well as abiotic factors such as salt, cold, and heat stresses.

**Conclusions:**

Both monocotyledonous and dicotyledonous CBP family proteins can be divided into two distinct groups, which suggest the possibility of functional diversification. The high similarities of protein sequences, conserved domains and three-dimensional models among OsHATs and their homologs in *Arabidopsis* and maize suggested that OsHATs have multiple functions. OsHAC701, OsHAG702, and OsHAG704 were localized in both the nucleus and cytosol in transient expression analyses with *Arabidopsis* protoplasts. *OsHATs* were expressed constitutively in rice, and their expression was regulated by exogenous hormones and abiotic stresses, which suggested that OsHATs may play important roles in plant defense responses.

## Background

Histone modification plays a key role in the regulation of gene expression [1]. Acetylation by histone acetyltransferases (HATs) is normally correlated with increased gene activity, whereas deacetylation via histone deacetylases (HDACs) is often associated with gene repression [2,3]. In eukaryotes, histone acetylation is catalyzed by five distinct HAT families, which comprise the p300/CREB (cAMP-responsive element-binding protein)-binding protein (CBP) family, the TATA-binding protein-associated factor (TAF)_II_250 family, the general control non-repressible 5-related N-terminal acetyltransferase (GNAT) family, the MOZ, Ybf2/Sas3, Sas2, and Tip60 (MYST) family, and the nuclear hormone-related HATs family [4,5]. Bioinformatics analysis suggests that currently there are 12 putative HATs in *Arabidopsis thaliana*, and these proteins belong to the CBP family (HAC1/PCAT2, HAC2/PCAT1, HAC4/PCAT3, HAC5/PCAT4, and HAC12), the TAF_II_250-related family (HAF1 and HAF2/TAF1), the GNAT family (HAG1/GCN5, HAG2, and HAG3/ELP3) and the MYST family (HAM1/HAG4 and HAM2/HAG5) [5,6].

In *Arabidopsis*, accumulating evidence indicates that HATs contribute to many aspects of plant growth and development, including root development [6,7], floral development [6,8,9], gametophyte development [10], and cell proliferation during organ growth [11]. In addition, histone acetylation by HATs is important for plant adaptation to environmental changes, such as light signaling [12-15], salt stress [16], cold stress [17-19], heat stress [20], abscisic acid (ABA) signaling [16,21,22], and other hormone signaling [23].

Rice is an economically important crop and a model plant for genomics and molecular biology research in monocotyledons. Eight HATs have been identified in rice (OsHATs) and these proteins can be grouped into four major families, namely the CBP (OsHAC701, OsHAC703, and OsHAC704), TAF_II_250 (OsHAF701), GNAT (OsHAG702, OsHAG703, and OsHAG704), and MYST (OsHAM701) families. The GNAT family can be further divided into three subfamilies, namely GCN5, ELP3 and HAT1 [5]. OsHAG702, OsHAG703, and OsHAG704 belong to the GCN5, ELP3, and HAT1 subfamilies, respectively [24]. Some phylogenetic analyses of HATs have been performed previously [5,10,24,25]. However, the evolutionary relationships of the CBP and TAF_II_250 families remain unclear. In addition, the biological functions of OsHATs in rice have not been addressed.

To investigate potential functions of OsHATs, systematic bioinformatics and expression analyses were performed. Phylogenetic trees for the CBP and TAF_II_250 families were generated to explore the evolutionary relationships among representative species of monocotyledons (monocots), dicotyledons (dicots), bryophytes, pteridophytes, animals and fungi. Multiple sequence alignment and domain analysis were used to predict the specific functions of OsHATs in comparison with the HATs of other organisms. We also generated three-dimensional (3D) comparative protein structure models of HATs with the SWISS-MODEL [26-28]. In addition, the subcellular localization of the eight OsHATs was predicted by protein sequence analyses. Transient expression of OsHAC701, OsHAG702, and OsHAG704 in *Arabidopsis* protoplasts was performed to determine the subcellular localization. Finally, the expression patterns of *OsHATs* were analyzed using real-time quantitative polymerase chain reaction (PCR) analysis (RT-qPCR). The results obtained will make an important contribution to the elucidation of the functions of different HATs in rice.

## Methods

### Searches of HAT cDNA and protein sequences

We searched for existing OsHATs sequence data in the NCBI ( http://www.ncbi.nlm.nih.gov/), ChromDB ( http://www.chromdb.org/), UniProt ( http://www.uniprot.org/), and KOME ( http://cdna01.dna.affrc.go.jp/cDNA/) databases. HAT cDNA sequences and protein sequences were downloaded from ChromDB ( http://www.chromdb.org/). The sequences represented the monocots *Oryza sativa* ( *japonica* cultivar group, Os), *Oryza sativa* ( *indica* cultivar group, Osi), *Zea mays* (Zm), *Sorghum bicolor* (Sb), and *Triticum aestivum* (Ta); the dicots *Arabidopsis thaliana* (At), *Populus trichocarpa* (Pt), and *Glycine max* (Gm); the bryophyte *Physcomitrella patens* (Pp) and pteridophyte *Selaginella moellendorffii* (Sm); the animals *Caenorhabditis elegans* (Ce), *Drosophila melanogaster* (Dm), and *Homo sapiens* (Hs); and the fungi *Saccharomyces cerevisiae* (Sc) and *Schizosaccharomyces pombe* (Sp). The ChromDB nomenclature for all HAT proteins is followed. The theoretical isoelectric point (pI) and molecular weight (Mw) of the rice HAT proteins were calculated with the Compute pI/Mw online tool ( http://web.expasy.org/compute_pi/) (Table 1).

**Table 1:** List of rice HAT proteins

**Protein group**	**ChromDB ID**	**UniProt accession**	**Chromosome**	**Molecular weight**	**Isoelectric point**
CBP family	HAC701	Q9XHY7	1	145097.97	6.81
	HAC703	Q6YXY2	2	188724.98	8.46
	HAC704	Q5Z8V7	6	189576.06	8.41
TAF_II_250 family	HAF701	Q67W65	6	204274.57	5.48
GNAT family	HAG702	Q338B9	10	56685.34	6.34
	HAG703	Q7X7L3	4	63775.32	8.82
	HAG704	Q6ES10	9	52113.03	4.94
MYST family	HAM701	Q8LI34	7	51104.13	7.22

### Phylogenetic analyses

Protein sequences were aligned with ClustalX 2.1 [29]. Unrooted radial trees were generated with the neighbor-joining method in conjunction with a bootstrap analysis of 1000 replicates using the PHYLogeny Inference Package (PHYLIP) version 3.6 [30]. The Dayhoff PAM model of protein evolutionary changes [31] was used to measure the branch lengths (evolutionary time) of the tree with the PROTDIST program. TreeView version 1.6.6 was used to display and edit the phylogenetic trees [32].

### Sequence analyses and alignments

Multiple sequence alignments of representative HAT proteins were generated with the ClustalW2 online tool ( http://www.ebi.ac.uk/Tools/msa/clustalw2/). Protein domains and function sites of all HAT proteins from rice and other organisms were assigned to the regions of target sequences with InterProScan [33] using the SWISS-MODEL Workspace website [26-28] ( http://swissmodel.expasy.org/workspace/index.php?func=tools_sequencescan1). UniProtKB/TrEMBL ( http://www.uniprot.org/blast/) was used to explore further the conserved domains and compositional biases in amino acid sequences [34]. DOG 1.0 was used for drawing protein domain structures [35]. UniProt BLAST ( http://www.uniprot.org/blast/) was used to determine the identities of OsHAT proteins and HAT proteins from other organisms [34]. 3D comparative protein structure models of HATs were generated with the automatic modeling mode of SWISS-MODEL [26-28] implemented on the SWISS-MODEL Workspace website ( http://swissmodel.expasy.org/). The protein structures were color-coded.

### Subcellular localization prediction

SLP-Local ( http://sunflower.kuicr.kyoto-u.ac.jp/~smatsuda/slplocal.html) is a subcellular location predictor based on local features of amino acid sequences [36,37]. TargetP version 1.1 ( http://www.cbs.dtu.dk/services/TargetP/) predicts the subcellular localization of eukaryotic proteins from the outputs of ChloroP and SignalP [38]. The protein subcellular localization predictor WoLF PSORT ( http://wolfpsort.org/) [39] is based on PSORTII and iPSORT. Nuclear localization signals (NLS) were discriminated by traditional PSORTII prediction [40] from the detailed results of WoLF PSORT. The NetNES 1.1 server ( http://www.cbs.dtu.dk/services/NetNES/) was used to predict leucine-rich nuclear export signals (NES) in OsHATs by a combination of neural networks and hidden Markov models [41].

### Plant materials and growth conditions

*Arabidopsis thaliana* cultivar Columbia-0 (Col-0) was used for protoplast isolation and transient expression analyses. Plants were grown in soil in a controlled-environment chamber with a long photoperiod (16 h light/8 h dark) at 22 ± 2°C.

Rice (*Oryza sativa* L. subsp. *japonica* cv. Nipponbare) seeds were imbibed with water in the dark at 37 ± 1°C for 24 h and then placed on filter paper (VWR International, Mississauga, ON, Canada) moistened with water in Petri dishes at 23 ± 1°C in the dark. After germination, rice seedlings were grown in beakers containing water, in a culture room with a daily photoperiodic cycle of 9 h light and 15 h dark. The culture room temperature was 23 ± 1°C.

For ABA (Sigma, Oakville, ON, Canada), salicylic acid (SA; Fisher Scientific, Ottawa, ON, Canada), and high salinity treatment, seedlings at the two-leaf stage growing in beakers were transferred to water with or without ABA (100 μM), SA (100 μM) or NaCl (300 mM). For cold treatment, rice seedlings were incubated at 4 ± 1°C in the dark for 3 h. For heat treatment, seedlings were incubated in a 42°C incubator in the dark for 3 h. Seedlings maintained in water at 23 ± 1°C in the dark were used as controls. Leaves of rice seedlings at the two-leaf stage were harvested after treatment, frozen in liquid nitrogen, and stored at −80°C.

### Protoplast isolation and transient expression

The full-length cDNAs of *OsHAC701* were subcloned into the p2YGW7.0 vector [42] to create the YFP-OsHAC701 construct, whereas the full-length cDNAs of *OsHAG702* and *OsHAG704* were subcloned into the pSAT6-EYFP-N1 vector [43] to generate the OsHAG702-YFP and OsHAG704-YFP constructs. The isolation and transfection of *Arabidopsis* leaf mesophyll protoplasts were conducted as previously described [44,45]. Briefly, protoplasts were isolated from well-expanded leaves of 3-week-old *Arabidopsis* plants. Volumes (10–20 μg) of the OsHAC701/HAG702/704-YFP fusion plasmid and VirD2NLS-mCherry as a nuclear marker [46] were cotransfected into 150 μl protoplasts (3 × 10^4^ protoplasts) using a PEG–calcium transfection solution. Protoplasts were incubated at 22 ± 2°C under white light overnight to allow expression of the introduced genes. The YFP fluorescence was examined and photographed using a Leica SP5 confocal microscope (Leica, Wetzlar, Germany).

### Real-time quantitative PCR analysis

Total RNA was extracted from leaves of rice seedlings at the two-leaf stage using a modified lithium chloride precipitation method [47]. The quantity and quality of RNA were measured using a Thermo Scientific NanoDrop^TM^ 1000 spectrophotometer (Wilmington, DE, USA). RNA was treated with DNase I (New England Biolabs, Pickering, ON, Canada) for 15 min prior to cDNA synthesis. cDNA was synthesized from 2 μg RNA with the ThermoScript^TM^ RT-PCR System (Invitrogen, Burlington, ON, Canada). RT-qPCR analysis was performed with the SsoFast^TM^ EvaGreen® Supermix (Bio-Rad, Mississauga, ON, Canada). Data were collected with the CFX96^TM^ Real-Time PCR Detection System (Bio-Rad) in accordance with the manufacturer’s instructions. RT-qPCR data were expressed as the cycle number necessary to reach a threshold fluorescence value (Ct) and analyzed with the comparative Ct method (ΔΔCt). The reported values were the means of three biological replicates, and each biological replicate consisted of three technical replications. *eEF-1α* (Eukaryotic elongation factor 1-alpha [48]) and *Ubq-1* (AK059011.1, Ubiquitin [49]) were used as reference genes to normalize the expression data. *PR10a* (Pathogenesis-related gene), which can be induced by SA or ABA [3,50], was selected as a control to determine whether the SA and ABA treatments were effective in this study. The primers for RT-qPCR are listed in Table 2. The specificity of primer pairs was confirmed by melt curve analysis in comparison with triplicate no-template controls. PCR amplification efficiency was calculated from a standard curve of Ct versus the logarithm of starting template quantity. Each assay was optimized so that the amplification efficiency ranged between 90% and 110%, and with a coefficient of determination (*R*^2^) greater than 0.980 (Table 2).

**Table 2:** Primer pairs used for RT-qPCR

**Gene name**	**Forward primer**	**Reverse primer**	**Amplification efficiency**	***R***^**2 **^**value**
*eEF-1α*	TTTCACTCTTGGTGTGAAGCAGAT	GACTTCCTTCACGATTTCATCGTAA	90.60%	0.993
*Ubq-1*	AACCAGCTGAGGCCCAAGA	ACGATTGATTTAACCAGTCCATGA	91.50%	0.993
*PR10a*	AAGTCATGTCCTAAAGTCGGATG	ATAGTAGCCATCCACGATGTCCT	107.80%	0.993
*HAC701*	TGGCGGTGCTTGGTTTGCCT	ACGGGCACGGGTATGACATCGT	106.80%	0.982
*HAC703*	TGTTGAAGAGGTGAAACGTGGG	GCTTCAACCGTTTAAAAAGCCGA	99.20%	0.985
*HAC704*	CAGTGACGAACCAGAGGAAGGGTG	AGGCATGCGCAAACCACGTT	109.90%	0.981
*HAF701*	ACCAGTGCCGCAGATGACGA	TCCGCCAGTGCAAAAAGGTGCT	109.70%	0.989
*HAG702*	TTGCTCGGCAGCTTCCTAACATGC	CAGCATCTCGGGCATGTTGCTTCA	99.50%	0.997
*HAG703*	TGCTGCAAATGAGGGCTGGGA	CGGCCACATTTTCGCAATCGCA	103.10%	0.985
*HAG704*	AAGCGGCTCGTCCAAATGCC	TTGCCGCGTGAGGTGACGTT	93.10%	0.996
*HAM701*	ACCGGAGCGCCCTCTTTCTGAT	AGAACCTTGGGGTCAGCGCA	99.80%	0.993

Amplification efficiency and coefficient of determination (*R*^2^ value) for each primer pair were determined from a standard curve by RT-qPCR.

All RT-qPCR data were expressed as the mean ± standard error. Statistical differences of expression of each *OsHAT* among rice tissues were assessed by one-way analysis of variance (ANOVA) followed by the least significant difference (LSD) and Student-Neumann-Keuls (SNK) post hoc comparison. The analyses were performed with SPSS 13.0 software (SPSS Inc., Chicago, IL, USA). The threshold of significance was defined as *p* < 0.05. Student’s *t*-test was used to assess the significance of differences between the exogenous treatment and the control. Significance was established at *p* < 0.05 or *p* < 0.01.

### Western blot analysis

Extraction of acid-soluble proteins was performed based on the descriptions by Tariq et al. [51] and Probst et al. [52]. Fresh rice leaves (0.3 g) were ground in liquid nitrogen and homogenized in 2.25 ml freshly prepared extraction buffer using a Fisher Scientific Model 100 Sonic dismembrator. After centrifugation (15 min, 20,000 rcf, 4°C, twice), the supernatant was stored at −80°C. Protein concentration was determined by the Micro-Bradford Assay with the Bio-Rad Protein Assay Solution. Protein extracts were added to 18.5 mM dithiothreitol, separated on a 16% sodium dodecyl sulfate polyacrylamide electrophoresis gel, and transferred to a polyvinylidene fluoride membrane using a Bio-Rad Semi-dry electrophoretic transfer cell (15 V, 15 min). The following antibodies were used: anti-Histone H3 (1:7500 dilution; Cell Signaling Technology, Inc., Danvers, MA, USA), anti-acetyl-Histone H3K18 (1:10,000 dilution; Cell Signaling), anti-acetyl-Histone H3K9 (1:1000 dilution; Cell Signaling), and anti-acetyl-Histone H4K5 (1:20,000 dilution; EMD Millipore, Billerica, MA, USA). Histone H3 was applied as an equal loading control. The bound immune-complexes were detected with ECL Plus western blot detection reagents (GE healthcare Life Sciences, Baie-d'Urfé, QC, Canada) and exposed to Classic Single-Emulsion Autoradiography Film (Mandel Scientific, Guelph, ON, Canada). The films were developed automatically with an AGFA CP1000 X-Ray Film Processor and scanned with an UMAX Powerlook 1120 scanner.

## Results and Discussion

### CBP family of HATs

**Figure 1:**
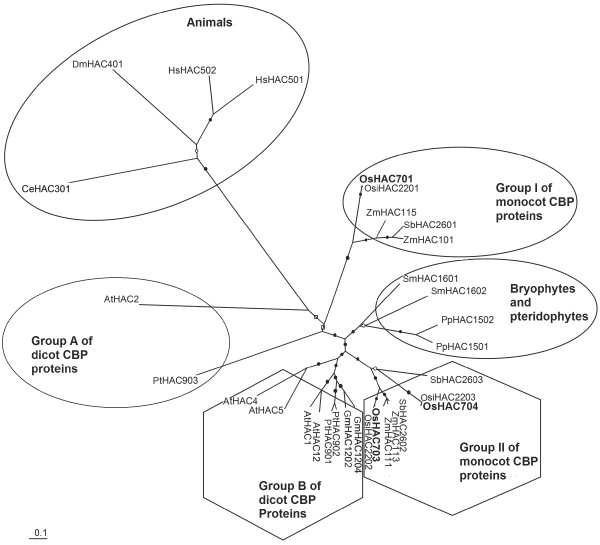
**Phylogenetic analysis of CBP family proteins from different plant and animal species.** An unrooted tree was constructed using the neighbor-joining distance method with the PHYLIP package. Bootstrap support values (1–1000) for individual branches are indicated as: excellent support (bootstrap value ≥ 995; filled circle); strong support (bootstrap value > 910; empty circle); and good support (bootstrap value = 660; empty square).

Three CBP family proteins were identified in rice, namely OsHAC701, OsHAC703, and OsHAC704. Phylogenetic analysis of the CBP family proteins from different plant and animal species (Figure 1) showed that the monocot CBP proteins could be divided into two distinct groups (Group I and Group II). Group I contained OsHAC701, whereas Group II contained OsHAC703 and OsHAC704. Likewise, the dicot CBP proteins were grouped into two distinct groups (Group A and Group B). Further analysis (see Additional file 1) indicated that OsHAC701 was only 46.0% and 42.0% identical with OsHAC703 and OsHAC704, respectively. By contrast, OsHAC703 and OsHAC704 shared 80.0% sequence identity. In addition, OsHAC701 on average showed 65.3% sequence identity to the monocot CBP proteins of Group I (SbHAC2601, ZmHAC101, and ZmHAC115; see Additional file 1), whereas on average it showed only about 43.0% sequence identity to monocot CBP proteins of Group II (SbHAC2602, SbHAC2603, ZmHAC111, and ZmHAC113). These observations further reinforced the results of the phylogenetic analysis that rice CBP proteins could be divided into two groups. Furthermore, OsHAC701, OsHAC703, and OsHAC704 were about 41.0%, 46.0% and 45.5% identical with Group A dicot proteins, and about 42.9%, 50.1% and 50.0% identical with Group B dicot proteins, which indicated that the Group II proteins were more similar to the Groups A and B proteins than to the Group I proteins. Rice CBP proteins showed low sequence identities with bryophyte proteins (PpHAC1501 and PpHAC1502) and pteridophyte proteins (SmHAC1601 and SmHAC1602), and even lower identities with animal proteins (HsHAC501, HsHAC502, DmHAC401, and CeHAC301). Taken together, these sequence analysis results (see Additional file 1) were consistent with the clusters obtained in the phylogenetic tree (Figure 1).

**Figure 2:**
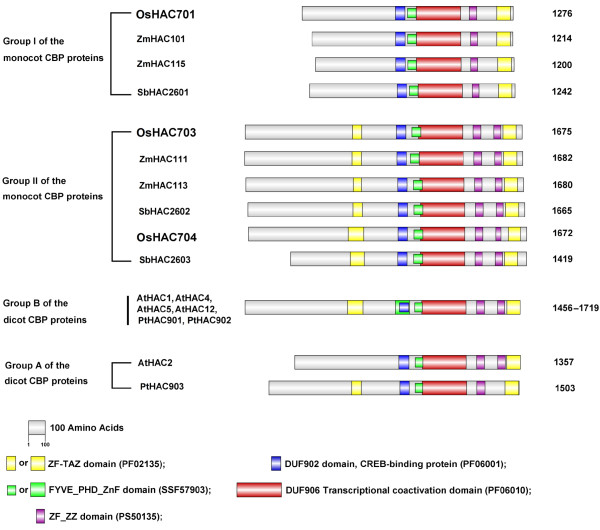
**Domain architecture of CBP family proteins in monocots and dicots.** The domain analysis was performed with InterProScan implemented in the SWISS-MODEL Workplace. The names and groups of the CBP proteins are indicated on the left of the figure. The protein lengths are displayed on the right. Different domains are represented by different colors and lengths at their precise position in the protein sequence from the N-terminus to the C-terminus. The Pfam accession number for the domains is shown in parentheses. The bioinformatics tool DOG 1.0 was used for this figure [35]. Abbreviations for organisms are: *Oryza sativa* subsp *. japonica* (Os), *Zea mays* (Zm), *Sorghum bicolor* (Sb), *Arabidopsis thaliana* (At), *Populus trichocarpa* (Pt).

Five domains (Figure 2), comprising the DUF902_CREB-binding protein domain (PF06001, residue range 565–622), FYVE/PHD-type zinc finger (SSF57903, 639–690), DUF906_transcriptional coactivation domain (PF06010, 691–958), ZZ-type zinc finger (PS50135, 1055–1177), and TAZ-type zinc finger (PF02135, 1177–1260), were identified in OsHAC701 and other Group I monocot CBP proteins (ZmHAC101, ZmHAC115, and SbHAC2601) with InterProScan [33]. By contrast, two additional domains were identified in OsHAC703 and OsHAC704 as well as other Group II CBP proteins; one was a ZZ-type zinc finger located near the C-terminal region between the ZZ-type zinc finger and the TAZ-type zinc finger, and the other was a TAZ-type domain displayed on the left side of the DUF902_CREB-binding protein domain. Similar to the Group II monocot proteins, the Group B dicot proteins all contained seven domains, but with one additional FYVE/PHD-type zinc finger that overlapped with the DUF902_CREB-binding protein domain. The DUF902 domain is found in several transcriptional coactivators related to transcriptional activation and histone acetylation [53]. The DUF906 domain involved in transcriptional coactivation is part of the CBP-type HAT domain [5]. TAZ-type, ZZ-type and FYVE/PHD-type zinc fingers have important roles in protein recognition and protein–protein interactions [54,55]. Evidence also suggests that PHD fingers interact with histones and other histone-recognition proteins [56]. These analyses indicated that OsHAC701, OsHAC703, and OsHAC704 may have HAT activity and transcription cofactor activities. The DUF902 and DUF906 domains are highly conserved within the CBP family proteins of plants. Members of plant CBP family proteins differ in the type and number of zinc fingers. Different types of zinc fingers may bind to different proteins or histones [54] and be recruited to different target areas of chromatin.

In terms of amino acid compositional bias, OsHAC701 contains a Ser-rich region (residue range 234–247) and a Cys-rich region (1130–1157). OsHAC703 contains two Gln-rich regions (111–135 and 402–625), whereas OsHAC704 contains a Poly-Ser stretch (132–135) as well as a Gln-rich region (379–435). A Cys-rich region in OsHAC701 implies that this protein may interact with other proteins [57]. Gln-rich regions in OsHAC703 and OsHAC704 may mediate transcriptional activation [58,59]. Similar to OsHAC703, AtHAC1, AtHAC4 and AtHAC12 all contain two Gln-rich regions, whereas AtHAC5 contains one Gln-rich region similar to OsHAC704 and AtHAC2 contains a Ser-rich region similar to OsHAC701.

AtHAC1 shows acetyltransferase activity [60] and transcriptional coactivator function for a heat-shock-inducible gene in a protoplast system [20]. Furthermore, AtHAC1, AtHAC5 and AtHAC12 have broad-specificity HAT activities and likely act together to acetylate histone H3 lysine 9 (H3K9) [61]. *In vivo*, AtHAC1, AtHAC5 and AtHAC12 play redundant roles in the promotion of flowering by repressing the expression of the floral repressor *FLC* (*FLOWERING LOCUS C*) [9]. Similarly, AtHAC1 was reported to regulate flowering time by epigenetic modification of factors upstream of *FLC *[6]. Furthermore, AtHAC1 interacts with a tomato heat stress transcription factor HsfB1 *in vitro* and *in vivo *[20]. It remains to be determined whether rice and *Arabidopsis* homologs of CBP family proteins have similar biological functions.

### TAF_II_250 family of HATs

**Figure 3:**
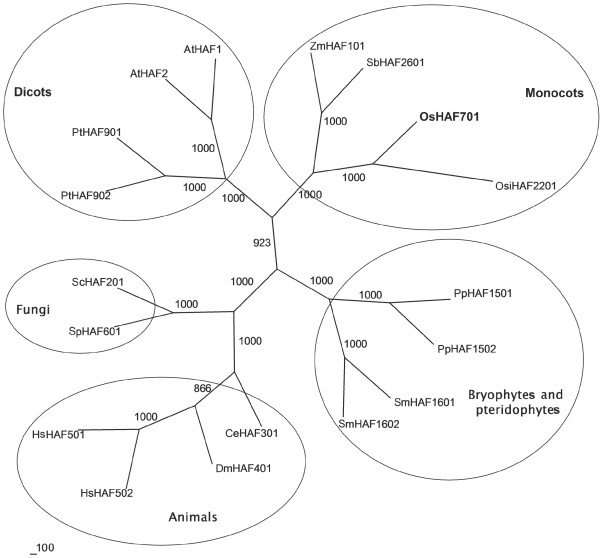
**Unrooted consensus tree of 18 TAF**_**II**_**250-type proteins.** Distinct plant (bryophytes, pteridophytes, monocots and dicots), fungus and animal clusters are resolved. Bootstrap support values (1–1000) for individual branches are shown. Clusters with a bootstrap value exceeding 700 are generally accepted to have a common origin (ancestor). In this figure, all bootstrap values are higher than 865.

OsHAF701 is the only TAF_II_250 family HAT in the rice genome. Other species (Figure 3) have one or two TAF_II_250-type proteins. Phylogenetic analysis of 18 TAF_II_250 family proteins showed that these proteins could be grouped into five clusters (Figure 3). Given the high bootstrap support for the tree topology, we deduced that all of the TAF_II_250 family proteins might share a common origin and belong to the same class. OsHAF701 was placed in the monocot cluster in the unrooted neighbor-joining tree. At the amino acid level, UniProt BLAST analysis revealed that OsHAF701 (see Additional file 2) had about 97.0% sequence identity with OsiHAF2201, 75.5% with other monocot homologs (Zm and Sb), 46.3% with dicot homologs (At and Pt), 41.0% with bryophyte and pteridophyte homologs (Pp and Sm), 29.8% with animal homologs (Dm, Ce and Hs), and 28.0% with fungus homologs (Sc and Sp).

**Figure 4:**
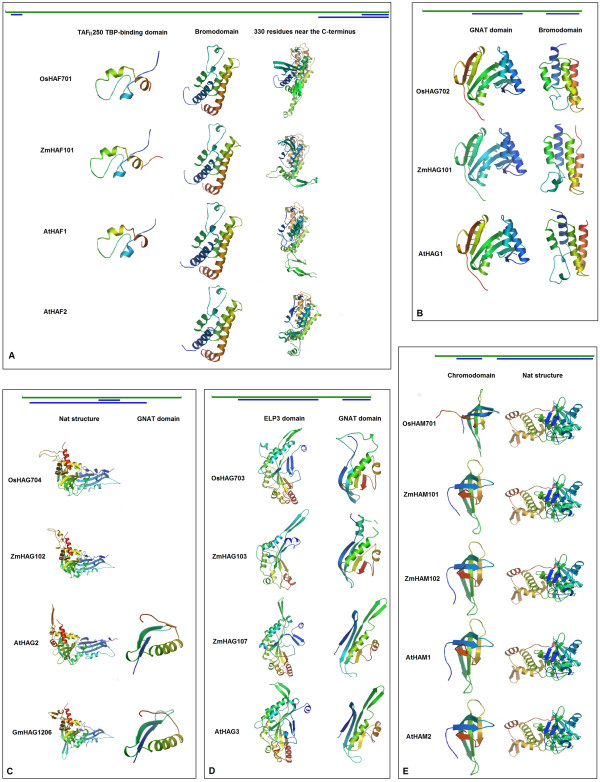
**Ribbon diagrams of the major 3D structures of HATs generated with SWISS-MODEL.** Green bars above the ribbon diagrams represent protein sequences with their corresponding amino acid lengths, and blue bars indicate the sequence areas where 3D structures were generated with SWISS-MODEL. Structures were color-coded ranging from the N-terminus (blue) to the C-terminus (red). ( **A**) TAF_II_250 family HATs from rice, maize and *Arabidopsis*. The SWISS-MODEL tool did not generate a 3D structure for the TAF_II_250 TBP-binding domain in AtHAF2. (**B**) GCN5 subfamily HATs. ( **C**) HAT1 subfamily HATs from rice, maize, *Arabidopsis* and soybean (Gm). The SWISS-MODEL tool did not generate a 3D structure for the GNAT domain in OsHAG704 and ZmHAG702. ( **D**) ELP3 subfamily HATs. ( **E**) MYST family HATs.

Domain analysis of TAF_II_250 family proteins in rice with InterProScan indicated that four major domains were present in OsHAF701: a TAF_II_250 TBP-binding domain (SSF47055, residue range 25–83), an ubiquitin domain (PF00240, 641–710), a C2HC zinc finger domain (1370–1388) and a bromodomain (PF00439, 1697–1779). Similar results are reported for *Arabidopsis*[5] and maize. Comparison of 3D models with SWISS-MODEL Workspace [26-28] revealed high similarities of 3D structures (especially two binding domains) existed among OsHAF701, AtHAF1, AtHAF2 and ZmHAF101 (Figure 4A).

In terms of amino acid compositional bias, OsHAF701 carried an Asp-rich region (18–110) in its N-terminal region, a Poly-Ala stretch (1004–1007) and a Poly-Lys stretch (1635–1645). AtHAF1 contained an Asp-rich region (16–106) near the N-terminus and a Poly-Lys stretch (1289–1296) near the C-terminus similar to OsHAF701. AtHAF2 contained two Poly-Lys stretches (999–1002 and 1195–1201).

TAF_II_250 family genes encode TATA-binding protein (TBP)-associated factor 1 [62]. The Asp-rich region responsible for substrate binding [63] overlapped with the TAF_II_250 TBP-binding domain in OsHAF701 and AtHAF1. The Asp-rich region may therefore participate in the regulation of enzyme activities [64,65]. A bromodomain known to bind to acetylated histone lysine residues [15,66] was identified in the C-terminal ends of both TAF_II_250 and GCN5 family proteins [5]. Sequence alignment showed conservation in the sequences of bromodomains within OsHAF701 and OsHAG702 (see Additional file 3A). In *Arabidopsis*, both AtHAF2/AtTAF1 and AtHAG1/AtGCN5 are required for H3K9, H3K27, and H4K12 acetylation of light-regulated genes involved in the light regulation of growth and development [12,15,62]. In addition, AtHAF2 regulates the expression of several cold-regulated genes independent of its HAT activity [19]. For AtHAF1, RNAi-mediated gene silencing of *AtHAF1* in *Arabidopsis* confers resistance to *Agrobacterium*-mediated transformation [67]. The similarities in the conserved domains and 3D models of OsHAF701, AtHAF1 and AtHAF2 indicate that OsHAF701 may have similar functions to those of AtHAF1 and AtHAF2.

### GNAT family of HATs

#### GCN5 subfamily of the GNAT family

A GCN5 homolog identified in rice is OsHAG702. Two domains are present in OsHAG702, namely a GCN5-related N-acetyltransferase (GNAT) domain (PS51186, residue range 168–315) and bromodomain (PF00439, 402–491). The 3D models of the GNAT domain and bromodomain analyzed with SWISS-MODEL Workspace [26-28] showed that high similarities existed among OsHAG702, AtHAG1 and ZmHAG101 (Figure 4B). As detected with UniProtKB, OsHAG702 carried three amino acid compositional biases in its N-terminal region (see Additional file 3B): a Ser-rich region (residues 8–67), a Poly-Asp stretch (68–73), and an Ala-rich region (104–124). Similar amino acid compositional biases were identified in other monocot GCN5 subfamily proteins such as ZmHAG101. AtHAG1 also contained a Ser-rich (3–66) region in its N-terminal region.

The Ser-rich region is implicated in the regulation of enzyme activities [57]. The Ala-rich region is essential for transcriptional repression and interaction with TBP (TATA-box binding protein) [68]. Consistent with the analyses of 3D models, alignment of the GCN5 subfamily proteins indicated that the GNAT domain and bromodomain were highly conserved in the GCN5 subfamily proteins of spermatophytes [24]. These results suggest that OsHAG702 might have similar functions to those of AtHAG1 and ZmHAG101. AtHAG1/AtGCN5 shows HAT activity [17], acetylate primarily H3K14 [61], and contribute to cold-regulated gene expression [17-19]. AtHAG1 is also involved in controlling floral meristem activity through regulation of the expression of *WUSCHEL* and *AGAMOUS*[8]. Furthermore, AtHAG1 is required for light-inducible gene expression [8,12-15], and is essential for root stem-cell niche maintenance [7] and the regulation of miRNA accumulation at both transcriptional and posttranscriptional levels [69]. Taken together, these data suggest that AtHAG1 is involved in both long-term epigenetic regulation of chromatin modification and short-term control of transcriptional switches. Given that OsHAG702 displays a high sequence similarity with AtHAG1, OsHAG702 may also play an important role in different aspects of plant development and stress response.

#### HAT1 subfamily of the GNAT family

Multiple sequence alignments show that the protein sequence of OsHAG704 has high sequence similarity with other HAT1 subfamily proteins in monocots and dicots (see Additional file C3). Domain analysis with InterProScan and 3D protein structure analysis indicated that rice and five other angiosperm HAT1-type proteins all contained a homologous structure called acetyl-coenzyme A: amino acid N-acetyltransferases (Nat) (SSF55729) of about 335 amino acid residues in length. A GNAT domain (PF00583), which was identified in AtHAG2 and GmHAG1204 by domain analysis with InterProScan, was not identified in other HAT1 subfamily proteins. A portion of the GmHAG1206 sequence (61 residues in length) forms a 3D ribbon diagram that is very similar to the GNAT domain of AtHAG2 (Figure 4C). On the basis of the multiple sequence alignment, the GNAT domain sequence in GmHAG1204 showed strong sequence similarity with that of the GmHAG1206 sequence (see Additional file 3C), which suggested that a GNAT domain is present in GmHAG1206. Furthermore, owing to the highly conserved sequences within the HAT1 subfamily proteins of six angiosperms (see Additional file 3C), we predict that OsHAG704 may also contain a GNAT domain.

The high similarities of protein sequences and 3D models among the monocot and dicot homologs of the HAT1 subfamily also suggested that they may have similar functional roles. AtHAG2 (a homolog of OsHAG704) acetylates histone H4K12 [61] and the expression of *ZmHAG102*/ *HAT-B* is repressed by ABA treatment during maize seed germination [70].

#### ELP3 subfamily of the GNAT family

A high sequence similarity was observed among the ELP3 subfamily proteins in plants [24], fungi and animals. Analyses of 3D models of ELP3 (TIGR01211) and GNAT (PS51186) domains in OsHAG703, ZmHAG103, ZmHAG107, and AtHAG3 with SWISS-MODEL [26-28] indicated high similarity of 3D structures existed among these proteins (Figure 4D). Sequence alignment (see Additional file 3D) and 3D structure analysis (Figure 4B, C, D) indicated that the GNAT domain in the ELP3 subfamily (OsHAG703) was different from those in the GCN5 subfamily (OsHAG702) and HAT1 subfamily (OsHAG704).

OsHAG703 and its homologs of the ELP3 subfamily may also have similar functions, because they shared high similarities in sequences and 3D structures. AtHAG3/AtELP3 is a member of the conserved Elongator HAT complex that interacts with RNA Pol II during transcript elongation and plays a role in cell proliferation during organ growth [11]. Furthermore, AtHAG3 can regulate plant response to ABA, oxidative stress resistance, and anthocyanin biosynthesis [21,22]. *Arabidopsis* Elongator also regulates the auxin signaling gene *SHY2/IAA3*, the auxin influx carrier *LAX2*, ethylene signaling, jasmonic acid signaling, and abiotic stress [23]. In addition, RNAi lines of *AtHAG3* are resistant to *Agrobacterium*-mediated transformation [67].

### MYST family of HATs

The MYST family is divided into five unrelated classes. Class I comprises proteins from the green lineage comprising the MYST family proteins of *Arabidopsis* and rice [10]. OsHAM701 was the only MYST family protein identified in rice. As shown in Figure 4E, the 3D structure of the chromodomain in OsHAM701 was similar to those in AtHAM1, AtHAM2, ZmHAM101, and ZmHAM102. Moreover, the 3D structure of the Nat structure in OsHAM701 was strikingly similar to those in AtHAM1, AtHAM2, ZmHAM101, and ZmHAM102. In addition, OsHAM701 contains an Ala-rich region and a Poly-Gly stretch at its N-terminus (see Additional file 3E).

Amino acid sequences of MYST-type proteins are highly conserved within monocots and dicots [24]. The Nat structure is the main conserved region for acetyltransferase activity. The chromodomain in the MYST family of HATs, similar to the bromodomain in HATs, is reported to be able to identify and bind specific histone residues [71]. The high similarities of amino acid sequences and 3D models of MYST-type HATs suggested that OsHAM701 might have a similar function to those of other MYST-type proteins in *Arabidopsis* and other angiosperms. AtHAM1 and AtHAM2 preferentially acetylate H4K5 [61] and are involved in gamete formation in both male and female organs in *Arabidopsis *[10].

### Subcellular localization prediction

Five subcellular localization prediction programs were used to determine the possible localization sites of HATs from rice (Table 3), *Arabidopsis* and maize (see Additional file 4). The subcellular localization of proteins with SLP-Local [36,37] generated localization sites for seven OsHATs with a low reliability index (RI) except for OsHAF701, which might be localized in the nucleus or cytosol with higher reliability. Similar SLP-Local results were obtained for AtHATs and ZmHATs. Only AtHAF1, AtHAF2, and ZmHAF101 generated a higher RI for the predicted subcellular localization (nucleus or cytosol). Likewise, TargetP [38] detected possible localization sites for most HATs with low reliability; however, OsHAF701, AtHAF2, ZmHAC115, and ZmHAF101 were predicted to be localized in the nucleus or cytosol with the strongest confidence (reliability class (RC) 1). WoLF PSORT [39] also showed low frequency values for HATs; however, OsHAC701 scored relatively high frequencies (7.5 out of 11 in the data set) for both nuclear and cytosolic localizations. In addition, AtHAC1 and ZmHAC101 received relatively high RI scores for both nuclear and cytosolic localizations. These results suggested that OsHAC701, AtHAC1, and ZmHAC101 might be dually localized proteins that undergo nucleocytosolic transport [72]. From the detailed results with WoLF PSORT, a traditional PSORTII predictor [40] identified NLS (pattern 4, pattern 7, or bipartite) in all OsHATs, AtHATs, and ZmHATs. Among OsHATs, OsHAF701 had the highest positive NLS score, suggesting the highest probability for nuclear localization, whereas OsHAM701 had a negative NLS score, which provided a strong indication for its cytosolic localization. NLSs were observed for all OsHAT proteins.

**Table 3:** Predicted subcellular localization of HATs from rice

**Protein**	**SLP-Local **^**a **^**(RI) **^**b**^	**TargetP **^**a **^**(RC) **^**c**^	**WoLF PSORT **^**d**^	**PSORTII (NLS score) **^**e**^	**NetNES: position-residue **^**f**^
OsHAC701	nucl or cyto(1)	mito(3)	nucl(13.5), cyto_nucl(7.5)	531aa, pat4; 419aa, pat7; (0.18)	1210-L, 1215-F
OsHAC703	nucl or cyto(1)	nucl or cyto(4)	nucl(14.0)	756, 1588aa, pat7; (0.22)	82-L, 83-A, 84-K, 85-R, 86-L, 87-E, 88-E, 89-I
OsHAC704	nucl or cyto(2)	nucl or cyto(3)	nucl(10.0), pero(2.0), mito(1.0)	71, 744aa, pat4; 741, 1585aa, pat7; (0.55)	51-I
OsHAF701	nucl or cyto(6)	nucl or cyto(1)	nucl(9.0), cyto(4.0)	259, 1069, 1266, 1523, 1641-1643aa, pat4; 251, 729, 1523, 1550, 1685, 1686aa, pat7; 1551, 1628aa, bipartite; (4.69)	1782-L, 1783-A, 1784-D, 1785-E, 1786-L, 1787-L, 1788-E, 1789-L
OsHAG702	Chlo(1)	chlo(4)	nucl(12.0), chlo(1.0)	22,23aa, pat4 NLS; (0.03)	323-L
OsHAG703	nucl or cyto(1)	nucl or cyto(5)	cyto(10.0), chlo(2.0), nucl(1.0)	17-19aa, pat4; 17aa, part7; (0.77)	414-L, 416-R, 417-M, 419-D, 422-L
OsHAG704	nucl or cyto(1)	nucl or cyto(4)	nucl(3.5), E.R.(3.0), cysk_nucl(2.5), chlo(2.0), plas(2.0), cyto(1.0), mito(1.0)	14-16aa, pat4; 14aa, pat7; (0.84)	183-L
OsHAM701	Chlo(1)	nucl or cyto(5)	nucl(6.0), cyto(4.0), plas(2.0), chlo(1.0)	374aa, pat4 NLS; (−0.29)	405-L

^a^: Subcellular locations predicted by SLP-Local and TargetP are chloroplast, mitochondria, secretory pathway, and other locations (nucleus or cytosol) for eukaryotic proteins.

^b^ RI: Reliability index ranges from 1 to 10. As the value of RI increases, the reliability of the SLP-Local prediction increases. chlo: chloroplast, the sequence contains a chloroplast transit peptide. nucl or cyto: any other location (nucleus or cytosol).

^c^ RC: Reliability class from 1 to 5. The lower the RC value is, the safer the prediction.

^d^ The numbers in parentheses indicate the prior probability that such protein localized to a given site is equal to the proportion of proteins in the data set [65]. Abbreviations and the number of proteins of the localization site in the data set: nucl: nucleus (456); chlo: chloroplast (750); cyto: cytosol (432); E.R.: endoplasmic reticulum (69); cysk_nucl: cytoskeleton and nucleus (0); plas: plasma membrane (165); mito: mitochondria (210); cyto_nucl: cytosol and nucleus (11); pero: peroxisomes (52). Last updated date: 2007/08/15.

^e^ Numbers reflect amino acid residues that showed nuclear localization signals (NLS). Three types of NLS (pat4, pat7, and bipartite) were detected in OsHATs. A positive NLS score indicates a higher probability of nuclear localization, and a negative NLS score indicates a higher probability for cytosolic localization.

^f^ The amino acid position and residue exhibiting predicted nuclear export signal (NES).

ZmHAG101/ZmGCN5 (a homolog of OsHAG702) contains a NLS in its N-terminus. Further studies confirmed that the N-terminus is responsible for nuclear targeting of ZmHAG101 [73]. Earley et al. [61] reported that AtHAG1 (a homolog of OsHAG702), AtHAG2 (a homolog of OsHAG704), and AtHAM1 and AtHAM2 (two homologs of OsHAM701) tended to be enriched at the periphery of the nucleolus, whereas AtHAM1 and AtHAM2 overlapped with the chromocenters containing the nucleolus organizer region, which suggested that these HATs might be important for the activation of ribosomal RNA genes. On the other hand, ZmHAG102/ZmHAT-B (a homolog of OsHAG704 and AtHAG2) is predominantly localized in the cytosol, and a significant proportion of ZmHAG102 is present in the nucleus [74]. NetNES [41] detected leucine-rich NES in all OsHATs as well as ZmHAG102. The presence of NES in all OsHATs suggests that they might be exported out of the nucleus.

### Nuclear and cytosolic localization of OsHAC701, OsHAG702 and OsHAG704

**Figure 5:**
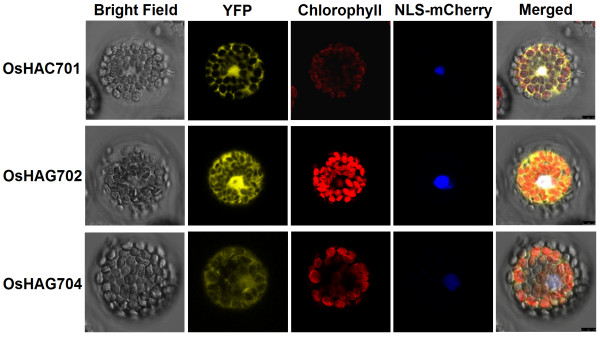
**Protoplast transient expression analyses using HAT-YFP fusion constructs.** Subcellular localization of OsHAC701, OsHAG702, and OsHAG704 was determined via *Arabidopsis* protoplast PEG transfection using HAT-YFP fusion constructs. OsHAC701 and OsHAG702 were localized in both the nucleus and cytosol. OsHAG704 was localized in the nucleus and cytosol, although YFP signals were relatively weak. Red color indicated autofluorescence emitted by chloroplasts. VirD2NLS-mCherry was used as a nuclear marker. Scale bars = 7.5 μm.

Transient expression analyses of OsHAC701/OsHAG702/OsHAG704-YFP fusion constructs in *Arabidopsis* protoplasts were used to determine the subcellular localization of OsHAC701, OsHAG702, and OsHAG704. As shown in Figure 5, OsHAC701-YFP, OsHAG702-YFP, and OsHAG704-YFP were localized in both the nucleus and cytosol. These experimental data were consistent with the subcellular localization predictions that OsHAC701, OsHAG702, and OsHAG704 all contain NLS and leucine-rich NES. Nuclear and cytosolic localization was also observed in the homolog of OsHAG704 in maize, ZmHAG102/ZmHAT-B [74]. HATs in humans catalyze acetylation of specific lysine residues not only in histone but also in non-histone substrates [75,76]. In *Arabidopsis*, it is suggested that lysine acetylation may occur on diverse proteins outside the nucleus [77,78]. The cytosolic localization of OsHAC701, OsHAG702, and OsHAG704 implies that these OsHATs might play an important catalytic role other than histone acetylation.

### Expression of *OsHATs* analyzed by RT-qPCR

#### Expression of OsHATs in different tissues

**Figure 6:**
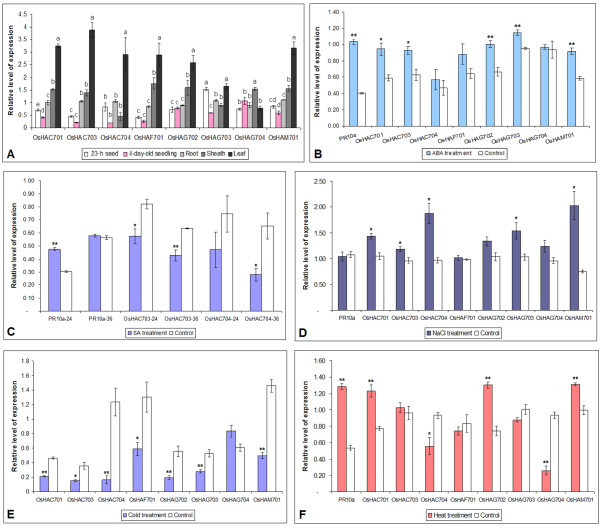
**Expression analyses of *OsHATs *in rice.** (**A**) RT-qPCR expression analyses of *OsHATs* in five different tissues: 23-h seed, seeds imbibed with water for 23 h; 4-day-old seedling, the whole plants of 4-day-old seedlings; and root, sheath and leaf tissues of two-leaf-stage seedlings. The relative amounts of mRNA for the eight *OsHATs* were measured. The data were expressed as the cycle number necessary to reach a threshold fluorescence value (Ct) and analyzed with the comparative Ct method (ΔΔCt). Expression values were normalized to those of *eEF-1α* and *Ubq-1.* Results are the average of three biological replicates, and each biological replicate consisted of three technical replications. Error bars represent standard errors. A different letter above each bar indicates a significant difference between tissues (*p* < 0.05, one-way ANOVA and LSD/SNK post hoc test). ( **B**- **F**) RT-qPCR analyses of *OsHATs* expression in rice leaves after treatment with hormones or abiotic stresses. The rice gene *PR10a* (known to be induced by ABA and SA) was used as a positive control. Expression was relative to *Ubq-1* gene expression. Color bars, treated plants; white bars, untreated plants. According to Student’s *t*-test, * and ** indicate a significant difference between the treatment and the control at *p* < 0.05 and *p* < 0.01, respectively. (**B**) Treatment of two-leaf-stage seedlings with 100 μM ABA for 24 h. The seedlings were kept in water for the same duration as the control. (**C**) Treatment with 100 μM SA for 24 h or 36 h. (**D**) Treatment with 300 mM NaCl for 12 h. (**E**) Treatment with 4 ± 1 °C in the dark for 3 h. (**F**) Treatment with 42 °C for 3 h.

RT-qPCR analysis was performed to examine the expression of *OsHATs* in five types of tissue, comprising seeds after 23-h imbibition, 4-day-old seedlings, and roots, sheathes, and leaves of two-leaf-stage seedlings (Figure 6A). Eight *OsHATs* were expressed in all tissues examined but with significant differences in the transcript abundance. The highest transcript levels of all *OsHATs* except *OsHAG704* were observed in the leaves of two-leaf-stage seedlings, whereas *O*s *HATs* transcript levels were very low in seeds after 23-h imbibition and 4-day-old seedlings. Constant expression in all of the tissues indicated that HATs play an important role in rice, but these proteins potentially have distinct functions with respect to development and defense against adverse conditions.

#### Expression of OsHATs in response to ABA treatment

The plant hormone ABA plays a pivotal role in a variety of developmental processes such as regulation of seed germination and seedling establishment [79]. ABA is also involved in plant responses to various stresses, such as salt, osmotic, cold, wound and pathogenic stresses [16,80,81]. To determine if the expression of *O*s *HATs* during rice seedling growth was regulated by ABA, we analyzed the expression pattern of *OsHATs* after treatment with 100 μM ABA for 24 h (Figure 6B). *PR10a* was used as a positive control. *PR10a* transcription increased by 2.55-fold after ABA treatment for 24 h, which indicated that the treatment was effective. Transcript levels of *OsHAC701*, *OsHAC703*, *OsHAG702*, *OsHAG703*, and *OsHAM701* were significantly elevated in response to exogenous ABA application. These results indicated that *OsHAC701*, *OsHAC703*, *OsHAG702*, *OsHAG703*, and *OsHAM701* may be involved in the ABA signaling pathway for response to environmental stresses during rice seedling growth.

Treatment with ABA for a short period of time (0–120 min) causes dynamic changes in histone H3 and H4 acetylation and phosphorylation in tobacco BY-2 cells, *Arabidopsis* T87 cells and whole leaves of tobacco and *Arabidopsis *[16]. Previous studies reveal that treatment of the leaves of rice seedlings with 100 μM ABA for 24 h increases the expression of *OsHDA702*, but represses the expression of *OsSRT701* and *OsSRT702 *[3]. ABA treatment of barley seedlings also induces the expression of *HvGCN5HvELP3*, and *HvMSYT *[24]. In contrast, ABA treatment of maize seeds for 48 or 72 h represses the expression of *ZmHAG101* (*ZmGCN5*) and *ZmHAG102* (*ZmHAT-B*), as well as several *ZmHDACs*. ABA selectively induces histone acetylation of the *VP1* gene (the embryogenesis-related gene *viviparous1*) and activates its transcription, which suggests that ZmHATs and ZmHDACs might participate in the ABA signal pathway during seed germination [70]. In *Arabidopsis* seedlings, ABA downregulates the expression of *AtHD2C*, an HD2-type *HDAC*, whereas overexpression of *AtHD2C* enhances the ABA tolerance of *Arabidopsis*, which suggests that AtHD2C may modulate ABA and stress responses [80]. Furthermore, the involvement of AtHAG3 (ELP3) in ABA responses in *Arabidopsis* is reported [21,22]. More recently, Chen et al. [82] reported that ABA treatment enriched the gene activation markers, histone H3K9K14 acetylation, and H3K4 trimethylation, but decreased the gene repression marker, H3K9 dimethylation, of a number of ABA-inducible genes in *Arabidopsis*. Taken together, these observations indicate that histone acetylation/deacetylation induced by HATs and HDACs is involved in the regulation of the ABA signaling pathway in a variety of plants including *Arabidopsis*, tobacco, maize, and rice.

#### Expression of OsHATs in response to SA treatment

SA plays an important regulatory role in multiple physiological processes such as the plant immune response. Furthermore, SA can interact with other phytohormones such as ABA, auxin, and gibberellic acid [83,84]. RT-qPCR analysis was performed using total RNA from leaves of two-leaf-stage seedlings exposed to 100 μM SA to examine whether *OsHATs* were regulated by SA (Figure 6C). The transcript level of *PR10a* increased by 1.58-fold in response to SA treatment for 24 h compared with that of the control plants. After SA treatment for 36 h, the expression levels of *OsHAC703* and *OsHAC704* were reduced to 67.43% and 42.76%, respectively, of those of control plants. No obvious changes were observed in the expression of the other six *OsHAT* genes after treatment with SA for 24 h or 36 h (data not shown). Previous studies show that treatment of the leaves of rice seedlings with 100 μM SA for 12 h downregulates the expression of *OsHDA702*, *OsHDA704*, *OsHDA706*, *OsSRT701*, and *OsSRT702* but upregulates the expression of *OsHDT702 *[3]. These observations indicate that members of the *HAT* and *HDAC* gene families may be involved in the SA signaling pathway in plant defense responses.

#### Expression of OsHATs in response to salt, cold and heat treatment

With salt treatment, the transcript levels of *OsHAC701*, *OsHAC703*, *OsHAC704*, *OsHAG703*, and *OsHAM701* were increased by 36.02%, 23.41%, 94.11%, 49.57%, and 170.32%, respectively, in comparison with the controls (Figure 6D).

The expression of eight *OsHATs* was also measured in seedlings exposed to low temperature. Except for *OsHAG704*, the expression of the other *OsHATs* was decreased to 45.84%, 42.20%, 13.32%, 45.17%, 34.81%, 53.05% and 34.16%, respectively, after 3 h of cold exposure, in comparison with the controls (Figure 6E).

In response to heat treatment (Figure 6F), *PR10a* transcripts increased by 2.39-fold in leaves of two-leaf-stage seedlings after exposure to 42°C for 3 h. Compared with the controls, the transcript levels of *OsHAC701*, *OsHAG702*, and *OsHAM701* were increased by 58.97%, 75.77% and 31.84%, respectively, in response to heat treatment. Conversely, the expression of *OsHAC704* and *OsHAG704* were decreased by 39.99% and 71.92%, respectively.

Previous research shows that salt stress induces the expression of *OsHDA702*, whereas the expression of *OsHDA704*, *OsHDA712*, and *OsSRT702* is decreased. Cold treatment represses the expression of *OsHDA704*, *OsHDA712*, and *OsSRT701* but induces the expression of *OsHDA702*[3]. AtHAG1 contributes to the expression of cold-regulated genes during cold acclimation [17-19]. AtHAF2 participates in the regulation of some cold-regulated genes [19]. AtHAC1 interacts with a tomato heat stress transcription factor HsfB1 that contains a histone-like motif [20]. In addition, an analysis of microarray data with Genevestigator indicated that heat stress (38°C for 3 h) upregulates the expression of *AtHDA6*, *AtHDA7*, *AtHDA5*, *AtHDA8*, and *AtHDA14*[72]. Taken together, HATs and HDACs can be modulated by salt, cold or heat stress, which suggests that these proteins may play an important role as epigenetic regulators for plant response to abiotic stress conditions.

With regard to the leaves of two-leaf-stage rice seedlings treated with salt for 12 h, western blot analysis showed that histone H3K18 acetylation was increased (see Additional file 5B), but no changes in the acetylation of H3K9 and H4K5 (Additional file 5C and D) were observed. Previous studies show that in cultured cells and leaves of *Arabidopsis* and tobacco, histone H3 Ser-10 phosphorylation, H3 phosphoacetylation and histone H4 acetylation are upregulated by salt treatment [16]. We observed increased transcript levels of *OsHAC701*, *OsHAC703*, *OsHAC704*, *OsHAG703*, and *OsHAM701* in response to salt stress, which was correlated with the increase in histone H3K18 acetylation. Therefore, these proteins may have a role in salt stress response in rice.

## Conclusions

The present phylogenetic analyses of the CBP and TAF_II_250 HAT family provide insights into the evolutionary relationships of these two protein families. Both monocot and dicot CBP family proteins can be subdivided into two distinct groups. Diversity in the specific domains identified in different OsHATs indicates that OsHATs have undergone functional diversification. The high similarities of protein sequences, conserved domains and 3D models among OsHATs and their homologs in *Arabidopsis* and maize suggests that OsHATs perform multiple functions during rice growth and development. Subcellular localization predictions indicate that all OsHATs might localize in both the nucleus and cytosol. Transient expression analyses of *Arabidopsis* protoplasts confirmed the nuclear and cytosolic localization of OsHAC701, OsHAG702, and OsHAG704. RT-qPCR analyses show that *OsHATs* are expressed constitutively in rice. In addition, their expression is modulated by exogenous treatment with the hormones ABA and SA as well as salt, cold and heat stresses, which suggests that OsHATs may play important roles in plant defense responses.

## Authors’ contributions

XL carried out the experiments, performed the bioinformatics analyses, and drafted the manuscript. ML performed the protoplast transfection and confocal microscopy. WZ and J Zhang participated in the RT-qPCR analysis. J Zhao participated in construction of the HAT-YFP fusion vector. LT, KW, and JD conceived of the project and acquired the funding. KW in discussion with LT and JD designed the study. LT, KW, and JD revised the manuscript. All authors read and approved the final manuscript.

## Supplementary Material

Additional file 1 Sequence identity analysis of CBP family proteins from plants and animals using UniProt Blast. Click here for file

Additional file 2 **Sequence identities of OsHAF701 versus 17 TAF**_**II**_**250-type proteins using UniProt BLAST.** Click here for file

Additional file 3 **Amino acid sequence alignment of HATs in monocots and dicots using ClustalW2.** *, positions highly conserved; positions highly conserved; **.**, positions of low sequence similarity; blanks, variable positions. (A) Amino acid sequence alignment of bromodomains in OsHAG702 and OsHAF701. (B) Multiple sequence alignment of the N-terminus of the GCN5 subfamily proteins. Ser-rich regions are indicated by black shading, Poly-Asp stretches by yellow shading, Ala-rich regions by green shading, and Asp-rich regions by red type. (C) Multiple sequence alignment of the HAT1 subfamily proteins. The red line above sequences indicates the Acyl-CoA N-acyltransferase (Nat) structure. Sequences enclosed in the rectangle are distinct or uncertain GNAT domains. The Poly-Ser stretch in rice is highlighted with yellow shading. (D) Sequence alignment of GNAT domains in HAG702, HAG703, and HAG704. The predicted GNAT domain’s sequence in HAG704 has low similarity with the other two GNAT domains. (E) Amino acid sequence alignment of the N-terminus of the MYST-type proteins. Ala-rich regions in rice are shaded in gray, Poly-Gly stretches in rice are highlighted in red type, and the Pro-rich region in AtHAM2 is shaded blue. Click here for file

Additional file 4 **Predicted subcellular localization of HATs from *Arabidopsis *and maize.** Click here for file

Additional file 5 **Analysis of histone acetylation in response to salt treatment in rice leaves.** Leaves from two-leaf-stage rice seedlings treated with (Salt) or without (CK) 300 mM NaCl for 12 h were harvested. In the bottom panel of each figure, Coomassie blue staining shows equal protein loading. Histone H3 was used as a loading control (A). Western blot analysis was performed with the following antibodies: anti-Histone H3 (A), anti-acetyl-Histone H3K18 (B), anti-acetyl-Histone H3K9 (C) and anti-acetyl histone H4K5 (D). Data are representative of three independent experiments. Click here for file
