# Supplementary material for: Histone acetyltransferases in rice (Oryza sativa L.): phylogenetic analysis, subcellular localization and expression
Source: BMC Plant Biol. 2012 Aug 15;12:145. doi: 10.1186/1471-2229-12-145 (PMC3502346; doi:10.1186/1471-2229-12-145)
Supplement: Additional file 1 — Sequence identity analysis of CBP family proteins from plants and animals using UniProt Blast. [file 1471-2229-12-145-S1.doc]

| **Protein** | **Organism** | **UniProt**  **accession** | **UniProt identities (%)**  **Vs. OsHAC701 Vs. OsHAC703 Vs. OsHAC704** | | |
| --- | --- | --- | --- | --- | --- |
| OsHAC701 | *Oryza sativa* subsp*. japonica* | Q9XHY7 | 100.0 | 46.0 | 42.0 |
| OsHAC703 | *Oryza sativa* subsp*. japonica* | Q6YXY2 | 46.0 | 100.0 | 80.0 |
| OsHAC704 | *Oryza sativa* subsp*. japonica* | Q5Z8V7 | 42.0 | 80.0 | 100.0 |
| OsHAC2201 | *Oryza sativa* subsp*. indica* |  | 99.0 | 45.0 | 52.0 |
| OsHAC2202 | *Oryza sativa* subsp*. indica* |  | 46.0 | 94.0 | 80.0 |
| OsHAC2203 | *Oryza sativa* subsp*. indica* |  | 44.0 | 80.0 | 96.0 |
| SbHAC2601 | *Sorghum bicolor* | C5YQX8 | 62.0 | 45.0 | 44.0 |
| SbHAC2602 | *Sorghum bicolor* | C5XTZ4 | 43.0 | 79.0 | 64.0 |
| SbHAC2603 | *Sorghum bicolor* | C5Z9A0 | 44.0 | 53.0 | 51.0 |
| ZmHAC101 | *Zea mays* |  | 61.0 | 45.0 | 50.0 |
| ZmHAC111 | *Zea mays* |  | 42.0 | 74.0 | 79.0 |
| ZmHAC113 | *Zea mays* |  | 43.0 | 77.0 | 80.0 |
| ZmHAC115 | *Zea mays* |  | 73.0 | 46.0 | 50.0 |
| AtHAC1 | *Arabidopsis thaliana* | Q9C5X9 | 43.0 | 51.0 | 41.0 |
| AtHAC2 | *Arabidopsis thaliana* | Q9FYH1 | 38.0 | 40.0 | 40.0 |
| AtHAC4 | *Arabidopsis thaliana* | Q9LG11 | 43.0 | 50.0 | 47.0 |
| AtHAC5 | *Arabidopsis thaliana* | Q9LE42 | 43.0 | 44.0 | 39.0 |
| AtHAC12 | *Arabidopsis thaliana* | Q9FWQ5 | 42.0 | 50.0 | 41.0 |
| GmHAC1202 | *Glycine max* |  | 42.0 | 51.0 | 75.0 |
| GmHAC1204 | *Glycine max* |  | 43.0 | 49.0 | 74.0 |
| PtHAC901 | *Populus trichocarpa* | B9HG17 | 48.0 | 53.0 | 41.0 |
| PtHAC902 | *Populus trichocarpa* | B9N5C8 | 39.0 | 53.0 | 42.0 |
| PtHAC903 | *Populus trichocarpa* | B9H3R2 | 44.0 | 52.0 | 51.0 |
| PpHAC1501 | *Physcomitrella patens* | A9TAD6 | 40.0 | 45.0 | 38.0 |
| PpHAC1502 | *Physcomitrella patens* | A9U3C0 | 45.0 | 58.0 | 50.0 |
| SmHAC1601 | *Selaginella moellendorffii* | D8R0K0 | 37.0 | 54.0 | 46.0 |
| SmHAC1602 | *Selaginella moellendorffii* | D8SJY6 | 44.0 | 44.0 | 41.0 |
| HsHAC501 | *Homo sapiens* | Q92793 | 29.0 | 33.0 | 31.0 |
| HsHAC502 | *Homo sapiens* | Q09472 | 29.0 | 33.0 | 30.0 |
| DmHAC401 | *Drosophila melanogaster* | O01368 | 29.0 | 33.0 | 30.0 |
| CeHAC301 | *Caenorhabditis elegans* | P34545 | 28.0 | 29.0 | 33.0 |
