# Supplementary material for: Histone acetyltransferases in rice (Oryza sativa L.): phylogenetic analysis, subcellular localization and expression
Source: BMC Plant Biol. 2012 Aug 15;12:145. doi: 10.1186/1471-2229-12-145 (PMC3502346; doi:10.1186/1471-2229-12-145)
Supplement: Additional file 2 — Sequence identities of OsHAF701 versus 17 TAFII250-type proteins using UniProt BLAST. [file 1471-2229-12-145-S2.doc]

| **Protein** | **Organism** | **UniProt accession** | **UniProt identities (%)** |
| --- | --- | --- | --- |
| OsHAF701 | *Oryza sativa* subsp*. japonica* | Q67W65 | 100.0 |
| OsiHAF2201 | *Oryza sativa* subsp*. indica* | B8B0Q6 | 96.0 |
| ZmHAF101 | *Zea mays* |  | 75.0 |
| SbHAF2601 | *Sorghum bicolor* | C5Z6R1 | 76.0 |
| PtHAF901 | *Populus trichocarpa* | B9IJA8 | 50.0 |
| PtHAF902 | *Populus trichocarpa* | B9HED4 | 49.0 |
| AtHAF1 | *Arabidopsis thaliana* | Q8LRK9 | 45.0 |
| AtHAF2 | *Arabidopsis thaliana* | Q6PUA2 | 41.0 |
| PpHAF1502 | *Physcomitrella patens* | A9TKZ9 | 41.0 |
| PpHAF1501 | *Physcomitrella patens* | A9SMK3 | 39.0 |
| SmHAF1601 | *Selaginella moellendorffii* | D8RSQ1 | 41.0 |
| SmHAF1602 | *Selaginella moellendorffii* | D8RD05 | 43.0 |
| DmHAF401 | *Drosophila melanogaster* | E1JJ72 | 31.0 |
| CeHAF301 | *Caenorhabditis elegans* | Q9XUL9 | 30.0 |
| HsHAF501 | *Homo sapiens* | B1Q2X3 | 28.0 |
| HsHAF502 | *Homo sapiens* | Q8IZX4 | 30.0 |
| ScHAF201 | *Saccharomyces cerevisiae* | P46677 | 29.0 |
| SpHAF601 | *Schizosaccharomyces pombe* | Q09813 | 27.0 |
