# Supplementary figures and images for: Histone acetyltransferases in rice (Oryza sativa L.): phylogenetic analysis, subcellular localization and expression
Source: BMC Plant Biol. 2012 Aug 15;12:145. doi: 10.1186/1471-2229-12-145 (PMC3502346; doi:10.1186/1471-2229-12-145)

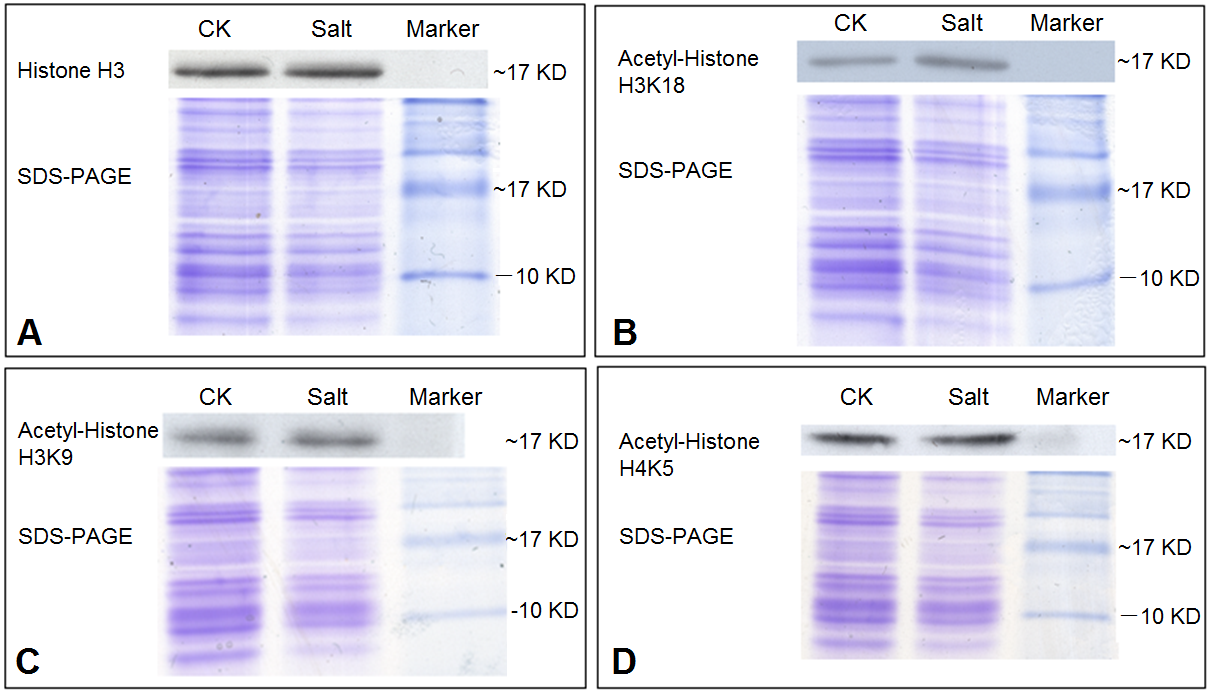

Supplement: Additional file 5 — Analysis of histone acetylation in response to salt treatment in rice leaves. Leaves from two-leaf-stage rice seedlings treated with (Salt) or without (CK) 300 mM NaCl for 12 h were harvested. In the bottom panel of each figure, Coomassie blue staining shows equal protein loading. Histone H3 was used as a loading control (A). Western blot analysis was performed with the following antibodies: anti-Histone H3 (A), anti-acetyl-Histone H3K18 (B), anti-acetyl-Histone H3K9 (C) and anti-acetyl histone H4K5 (D). Data are representative of three independent experiments. [file 1471-2229-12-145-S5.tiff]
