# Supplementary material for: Histone acetyltransferases in rice (Oryza sativa L.): phylogenetic analysis, subcellular localization and expression
Source: BMC Plant Biol. 2012 Aug 15;12:145. doi: 10.1186/1471-2229-12-145 (PMC3502346; doi:10.1186/1471-2229-12-145)
Supplement: Additional file 3 — Amino acid sequence alignment of HATs in monocots and dicots using ClustalW2. *, positions highly conserved; positions highly conserved; ., positions of low sequence similarity; blanks, variable positions. (A) Amino acid sequence alignment of bromodomains in OsHAG702 and OsHAF701. (B) Multiple sequence alignment of the N-terminus of the GCN5 subfamily proteins. Ser-rich regions are indicated by black shading, Poly-Asp stretches by yellow shading, Ala-rich regions by green shading, and Asp-rich regions by red type. (C) Multiple sequence alignment of the HAT1 subfamily proteins. The red line above sequences indicates the Acyl-CoA N-acyltransferase (Nat) structure. Sequences enclosed in the rectangle are distinct or uncertain GNAT domains. The Poly-Ser stretch in rice is highlighted with yellow shading. (D) Sequence alignment of GNAT domains in HAG702, HAG703, and HAG704. The predicted GNAT domain’s sequence in HAG704 has low similarity with the other two GNAT domains. (E) Amino acid sequence alignment of the N-terminus of the MYST-type proteins. Ala-rich regions in rice are shaded in gray, Poly-Gly stretches in rice are highlighted in red type, and the Pro-rich region in AtHAM2 is shaded blue. [file 1471-2229-12-145-S3.pdf]

A

```
OsHAG702   QLTNLMRSLLKNMNEHPDAWPFKEPVDSRDVPDYYDIIKDPIDLKTMSKRVESEQYYVTLEMFVADMKRMFSNAKTYNSPDTIYYKCASRLESFFSNKVASQLAQASTKN 110
OsHAF701   -LSNILEKIVDHLRTMSCSFLFRKPVTKKEAPDYFDIIERPMDLGTIRDKVR-KMEYKNREDFRHDVAQIALNAHTYNLN---RHPHIPPLADELLELCDYLLEESAD-- 103
            *:*::..::.::.   . ::  *::**  .::.***:***: *:**  *:  .:*.  :    *  .  * *    *:  ::    **:***         :    . *  .  :  :        *  :::
```

B

```
OsiHAG2201  -------------MDGLAAPSPSHS---GATSGGGASHRKRK-----------------------LPPSSLSDATAD---EDDDTTAPSSPSTSPSSPSRPSSPSSSHSDD--------DDD 72
OsHAG702    -------------MDGLAAPSPSHS---GATSGGGASHRKRK-----------------------LPPSSLSDATAD---EDDDTTAPSSPSTSPSSPSRPSSPSSSHSDD--------DDD 72
ZmHAG101    -------------MDGLVAPSPSHS---GATSGGGASHRKRK-----------------------LPPSSLSDATGD---EDDDTTAPSSPSTAPSSPSRPSSPSSSHSDD--------DDD 72
SbHAG2603   -------------MDGLAAPSPSHS---GATSGGGASHRKRK-----------------------LPPSSLSDATAD---EDDDTTAPSSPSTAPSSPSRPSSPSSSHSDD--------DED 72
PtHAG903    MDTHS-HLTAPNRSRSSQSPSPSHSASASASASATSSIHKRKLAAAAAA----YDDHLPP---FAPSSLSADTRDGALTSNDDLESISARGADSDSDDAEDSDTVLDDDEEDF----DND 108
PtHAG906    MDTHS-HLTAPNRSRSSQSPSPSH----SASASATSSIHKRKRAAAAAA----SDDHLPP---LAPSSFSADTRDGALTSNDDLESISARGADSDSDDAEDSDAVVDDDEDEF----DND 104
GmHAG1205   --MES-HGGGPLRSRSSQSPSPSH----SASASATSSIHKRKLAS---------EDHAPP---FPPSSFSADTRDGALTSNDDLESISARGADSDSDDDSE-DAVVDDDEDDYG--NDND 98
AtHAG1      MDSHSSHLNAANRSRSSQTPSPSH----SASASVTSSLHKRKLAATTAANAAASEDHAPPSSSFPPSSFSADTRDGALTSNDELESISARGADTDSDPDESEDIVVDDDEDEFAPEQDQD 116
                             .  :*****    .*::.  :* :***                     :.***:*  * *    .:*    . *.   : :...   .. .    .**          *:*

OsiHAG2201  DSLHTFTAARLDGAPP-----SSSGRPPKPESSTVSAAAAA-------AAAAAA--PKPDSASAAAG 125
OsHAG702    DSLHTFTAARLDGAPP-----SSSGRPPKPESSTVSAAAAA-------AAAAAA--PKPDSASAAAG 125
ZmHAG101    DSLHTFNAARLDGAPSG---GSASGRPPKPDSSSVSAAAAA-------AAAAVGGGPKPEPGSANAG 129
SbHAG2603   DSLHTFNAARLDGAPGG---GSASGRPPKPDSSSVSAASAA-------AAAAAGGGPKLEPGSATAG 129
PtHAG903    SSMRTFTTARLETTN--AAGSSSRNTKIKMENTNVKIENSDSGKDGGHTGTGA-VGPAAAGSSGPGI 172
PtHAG906    SSMRTFTAARLETTAGVSAAGSGRNAKIKIENTNVKIENSDSGKDGGHTGTGA-VGPAAAGSSAPGI 170
GmHAG1205   SSIRTFTASRLSNPP-----SAPRNTKLKTEISTVKIENSDGAKD---AVVGA-CAAASAG-SVPGI 156
AtHAG1      SSIRTFTAARLDSSS--GVNGSSRNTKLKTESSTVKLESSDGGKDGGSSVVGTGVSGTVGGSSISGL 181
            .*::**.::**. .         :  .   * : :.*.   :         : ...          *  .
```

C

```
OsiHAG2203  MALKQKGTDAAADPK---KRRRVGFSGIDAGVEANECMKVFIARNPDEAGSANSTSLQPFDLNHFFGEDGKIYGYKNLKINVWISAISFHAYADISFEETSDGGKGITDLKPVLQNIFGE 117
OsHAG704    MALKQKGTDAAADPK---KRRRVGFSGIDAGVEANECMKVFIARNPDEAGSANSTSLQPFDLNHFFGEDGKIYGYKNLKINVWISAISFHAYADISFEETSDGGKGITDLKPVLQNIFGE 117
ZmHAG102    MALKQKDTDAAATATGTKKRRRVFFSDTDAGVEANECMKVFLVWNPGEVSSVDCTAIQPFDLNHFFGEDGKIYGYKNLKINVWISAKSFHGYADVSFDETSDGGKGITDLKPVLQNIFGE 120
SbHAG2601   MALKQKGTDAAATDT--KKRRRVGFSGTDAGVEANECMKVFLVRNPDEVGSVDCTSIEPFDLNHFFGEDGKIYGYKNLKINVWISAKSFHGYADISFDETSDGGKGITDLKPVLQNIFGE 118
GmHAG1204   MGQKQRSSSDADNEV--KKRRRVGFSGVDSGVEAKDCITIYLVSSKEEFDAPESFVIHPVDLNSFFDDDGKIYGYEGLKITIWVSSISFYAYADITFQSSSDRGKGVTDLKSALQTIFAE 118
GmHAG1206   ------------------------------------------SSKEEFDAPESFVIHPVDLNSFFDDDGKIYGYEGLKITIWISSISFYAYADITFQSSSDRGKGVTDLKSALQTIFAE 77
PtHAG907    MGQKQQPTADPISEP--KKRRRVGFSNIDVGIEANQCILIYLVSSKEEVGASHSFRISPVDLNGFFDEDGKIYGYNGLKITIWVSSVSFHAFADIKFQSTSDGGKGITDLKSSLQRIFAD 118
AtHAG2      MVQKQQASAGPGTEP--KKRRRVGFSPADTGVEANECIKIYLVSSKEEVDSSDISSVKPVDLNDFFGDGGKIYGYQGLKINVWINSISLHSYADITYQSTINGDKGITDLKSALQNIFAE 118
                                                     .  * .: .   : *.*** **. *******:.***.:*:.: *::.:**:.::.: : .**:****. ** **.:

OsiHAG2203  NLVE-KDEFLKTFSKECEYLSNVVTDGNVIKHDASID--------EDSAVETRVQISYSISACKA-LLSLSNVTAEILFELNYKVLLLFFIVVWCHLFCFWLKASSTPIDITEHGWEMLL 227
OsHAG704    NLVE-KDEFLKTFSKECEYLSNVVTDGNVIKHDASID--------EDSAVEI---VRVELQGAAA-FLYCR------------LVPLILLLVEG-----------STPIDITEHGWEMLL 201
ZmHAG102    NLVE-KEEFLHTFSKECEYIRTAVTNGSAIKHDGSYE--------SDPAVEI---VRVELQGAAA-FLYSR------------LVPLVLLLVEG-----------STPIDIGEHGWEMLL 204
SbHAG2601   NLVE-KEEFLHTFSKECECIRTAVTNGSAIKLDGSYE--------SDPAVEI---VRVELQGAAA-FLYSR------------LVPLVLLLVEG-----------STPIDIGEHGWEMVL 202
GmHAG1204   TLVDSKDEFLQKYLVDNDFVRTNISNGEALKHKAFQGNISDYNPHTDSSTSTVEVVRLVAGNMTTGQLYSH------------LIPLTLLLVDG-----------SSPIDVTDSQWELYV 215
GmHAG1206   TLVDSKDEFLQKYLADNDFVRTNISNGETLKHKAFKENICDSNQLTDSSTSTVEVVRLVAGNMATGQLYSH------------LIPLTLLLVDG-----------SSPIDVTDSQWELYI 174
PtHAG907    TLVDNKDDFLQTFSTESHFVRSIISDGEILQHKTSNG-------HVDSNR----------NTAAGHLYSR------------LVPLVLLLIDG-----------SNPIDVDDPGWELYV 197
AtHAG2      TIVDTKDEFLQTFSTQRDFIRNMVSNGEVMHAGATDG----SSKNAEVVPSDPQVIRMEIGSPNAGLLYSR------------LVPLVLLFVDG-----------SNPIDVTDPDWHLYL 211
            .:*: *::**:.:  : . : . :::*. ::                :                   :  *                 : * ::::                 *.***: :   *.: :

OsiHAG2203  VVKKSAQASSSSNFLVLGFAAVHHFYHYPESTRLRISQILVLPPYQGEGHGLRLLETINSISESENIYDVTIEDPSDYLQYIRSSIDCLRLLTFDPIKPALCSMVSSLKDTNLSKRTSSL 347
OsHAG704    VVKKSAQASSSSNFLVLGFAAVHHFYHYPESTRLRISQILVLPPYQGEGHGLRLLETINSISESENIYDVTIEDPSDYLQYIRSSIDCLRLLTFDPIKPALCSMVSSLKDTNLSKRTSSL 321
ZmHAG102    VVKKATQEAGS-KFELLGFAAVHNFYHYPESIRLRISQILVLPPYQGEGHGLGLLEAINYIAQSENIYDVTIESPSDYLQYVRSSIDCLRLLMFDPIKPALGAIVLSLKETNLSKRAQSL 323
SbHAG2601   VVKKTTQEAVS-KFELLGFAAVHNFYHYPESTRLRISQILVLPPYQGEGHGLRLLEAINYIAQSENIYDVTIEDPSDYLQYVRSSIDCLRLLTFDPIKSALSAIVSSLKETNLSKRTHSL 321
GmHAG1204   LCQKKTDPQGEIQCRLIGFTAVYRFYHYPDDSRLRLSQILVLPPYQHKGYGRFLLEVLYDVAISENVFDFTVEEPLDHFQHVRTCVDSLRLLQFYPIQNIVTKAVSLLKQGKLSKKANCP 335
GmHAG1206   VCQKKTDQQGEIQYRLTGFTAVYRFYHYPDDSRLRLSQILVLPPYQHKGYGRFLLEVLYDVAISENVFDFTVEEPLDHFQRVRTCVDALRLLRFGPIQNIVTKAVSLLKQEKLSKKAHCP 294
PtHAG907    LIQKKSDEHGDTQHKLLGFTAVYRFYHYPDSTRLRLSQILVLPPYQHKGYGGHLVEVLSNVAILEDVYDLTVEEPLDYFQHVRTSIDKIRLLLFAPIQDAVNSTVSYLKQGKLSKKTHVP 317
AtHAG2      LIQKK-EEKEDPLYRIVGFTAIYKFYRYPDRLRMRLSQILVLPSFQGKGLGSYLMEVVNNVAITENVYDLTVEEPSEKFQHIRTCIDINRLRSFDPIKPDIDSAVQTLTKGKLSKKAQIP 330
            : :*   :    .    : **:*::.**:**:   *:*:*******.:* :* *   *:*.:  ::  *:::*.*:*.* : :* :*:.:*  **  * **:  :   *  *.. :***::

OsiHAG2203  KMVPPSDLAETVRQKLKINKKQFLRCWEILIYLNLDAEDRKSMDNFRACIYDRIKGEILGTSTGPNGKRLVQMPSNFDEETCFAVYWTQDGGDADDQTVEQQPEDLKTQEQQLNEVVDSQ 467
OsHAG704    KMVPPSDLAETVRQKLKINKKQFLRCWEILIYLNLDAEDRKSMDNFRACIYDRIKGEILGTSTGPNGKRLVQMPSNFDEETCFAVYWTQDGGDADDQTVEQQPEDLKTQEQQLNEVVDSQ 441
ZmHAG102    RMVPPADLMETVRQKLKINKKQFLRCWEILVFLSLDSQDHKSMDNFRACIYDRMKGEILGSASGTNRKRLLQMPTSFNKEASFAVYWTQEIEDEDEQTVEQQPEDLKTQEQQLNELVDIQ 443
SbHAG2601   RMVPPAELMETVRQKLKINKKQFLRCWEILIFLSLDSQEHKSMDNFRACIYDRIKGEILGSASGTNGKRLLQMVSSSN-EESFAVYWTQESGDADDQTVEQQPEDLKTQEQQLNELVDNQ 440
GmHAG1204   RLLPPPSAIEDVRKSLKINKQQFLQCWEVLIYIGLNPVD-KNTENFVSIILNRVKYDILGKDSGTSGKQLIEVPSDVDQEMSFVMFRSEANEAS----SVQMDDNQANQEEQLQRLVQER 450
GmHAG1206   RLLPPPSAIEDVRKSLKINKQQFLQCWEVLIYIGLNPVD-KNMENFVSIILNRVKYDILGKDSGTSGKQLIEVPSDVDQEMSFVMFRSEANEAS----TVQMDDNQANQEEQLQKLVQER 409
PtHAG907    CFNPPVSAVEDVRKTLKINRKQFLQCWEVLIYLGLDPVD-KYMEDFVAIISSRVKADVLGKDSGSSGKQVVEVPSDYNPEMSFVMFRSQDSEAA----RVWMDENQTNQEEQLQQLVDER 432
AtHAG2      RFTPPLNAIEKVRESLKINKKQFLKCWEILIYLALDPID-KYMEDYTSVITNHVRTDILGKDIETPKKQVVDVPSSFEPEASFVVFKSVNGEEAN--TNVQVDENKPDQEQQLKQLVEER 447
             : **  .   * **:.****::***:***:*::: *:. : *  ::: : * .::: ::**.    .   *::::: :. : * .*.:: :                   ::   **:**:.:*: :

OsiHAG2203  MEEIVEVAKNVTSRGKDKLSVSCSV 492
OsHAG704    MEEIVEIAKNVTSRGKDKLSVSCSV 466
ZmHAG102    IEEIAGVAKNVTSRCKDKMTELVVQ 468
SbHAG2601   MEEIIGVAKNVTSRGKDKLVELVAQ 465
GmHAG1204   VKEIQLIAEKVTLHLGSSGVVVN-- 473
GmHAG1206   VKEIQLIAEK--------------- 419
PtHAG907    VKEIKLIAQKVHHV----------- 446
AtHAG2      IREIKLVAEKVSKSGQTLKV----- 467
            :.**  :*::
```

D

```
OsHAG702    NDGVDEHMIWLVGLKNIFARQLPNMPKEYIVRLVMDRTHKSMMVIRNNIVVGGITYRPYTSQKFGEIAFCAITAD-----EQVKGYGTRL-MNHLKQHARDADGLTHFLTYADNNAVGYF 114
OsHAG704    --------FLVLGFAAVHH--FYHYPES--TRLRIS----QILVLP-----------PYQ----GE------------------GHGLRL-LE------------------------- 43
OsHAG703    --KCRDVRTREAGIQDIHHKIRPDEVELVRRDYAANEGWETFLSYEDT--QQDILIGLLRLRKCGRNVTCPELVGRCSIVRELHVYGTAVPVHGRDADKLQHQGYGTLLMEEAERIARKE 116
                      *:  :.     .  :        .     ::                       *.                     :*  : :.

OsHAG702    VKQGFTKEIT--LDKERWQGYIKDYDGGILMECRIDQ 149
OsHAG704    -------------------------------------
OsHAG703    HRSKKIAVISGVGTRHYYRKLGYELEGPYMVKCLV-- 151
```

E

```
PtHAM901    MGSIDTPKNPENGSTTLPPTTDASATYDGDQNPTNGMPPRVTEMTLPVESEVVKKRKTSM 60
PtHAM902    MGSIDTPKNPENGARTLPPATDAYATYEGDKN---GMPSRATEMTLPVESEAIKKRKASM 57
OsHAM701    MGSMEASTAPENGTAAAAAAAASTACNGAGGGGGAAAASNGGGVERRLRSSAASASWASH 60
OsiHAM2201  MGSMEASTAPENGTAAAAAAAASTVCNGAGGGGGAAAASNGGGVERRLRSSAASASWASH 60
ZmHAM101    MGSMEASTAPENGSSAAGGGAGGTACNGAG------AASNGGGMERRLRSSAASASWAAH 54
ZmHAM102    MGSMEASTAPENGS-AAGGGAG----NGAG------AASNGGGVERRLRSSAAPASWAAQ 49
TaHAM801    ---MELSTAPENGTAAAAAACN------GG------AAPANGGVERRLRSSAASASWAAH 45
AtHAM1      ---MGSSADTETAMIIATPASNHNNPATNGGDANQNHTSGAILALTNSESDASKKRRMGV 57
AtHAM2      ---MGSSANTETNGNAPPPSSNQKPPATNGVDGSHPPPPPLTPDQAIIESDPSKKRKMGM 57
                :  .  .*.                .    ..       .*.      .
```
